# Impaired Neurodevelopmental Genes in Slovenian Autistic Children Elucidate the Comorbidity of Autism With Other Developmental Disorders

**DOI:** 10.3389/fnmol.2022.912671

**Published:** 2022-06-23

**Authors:** Danijela Krgovic, Mario Gorenjak, Nika Rihar, Iva Opalic, Spela Stangler Herodez, Hojka Gregoric Kumperscak, Peter Dovc, Nadja Kokalj Vokac

**Affiliations:** ^1^Laboratory of Medical Genetics, University Medical Centre Maribor, Maribor, Slovenia; ^2^Department of Molecular Biology, Faculty of Medicine, University of Maribor, Maribor, Slovenia; ^3^Centre for Human Molecular Genetics, and Pharmacogenomics, Faculty of Medicine, University of Maribor, Maribor, Slovenia; ^4^Department of Animal Science, Biotechnical Faculty, University of Ljubljana, Ljubljana, Slovenia; ^5^Department of Pediatrics, University Medical Centre Maribor, Maribor, Slovenia

**Keywords:** reverse phenotyping, single event variants, NDD-associated genes, *GRIA1* gene, *NR4A2* gene, *SIN3B* gene

## Abstract

Autism spectrum disorders (ASD) represent a phenotypically heterogeneous group of patients that strongly intertwine with other neurodevelopmental disorders (NDDs), with genetics playing a significant role in their etiology. Whole exome sequencing (WES) has become predominant in molecular diagnostics for ASD by considerably increasing the diagnostic yield. However, the proportion of undiagnosed patients still remains high due to complex clinical presentation, reduced penetrance, and lack of segregation analysis or clinical information. Thus, reverse phenotyping, where we first identified a possible genetic cause and then determine its clinical relevance, has been shown to be a more efficient approach. WES was performed on 147 Slovenian pediatric patients with suspected ASD. Data analysis was focused on identifying ultrarare or “single event” variants in ASD-associated genes and further expanded to NDD-associated genes. Protein function and gene prioritization were performed on detected clinically relevant variants to determine their role in ASD etiology and phenotype. Reverse phenotyping revealed a pathogenic or likely pathogenic variant in ASD-associated genes in 20.4% of patients, with subsequent segregation analysis indicating that 14 were *de novo* variants and 1 was presumed compound heterozygous. The diagnostic yield was further increased by 2.7% by the analysis of ultrarare or “single event” variants in all NDD-associated genes. Protein function analysis established that genes in which variants of unknown significance (VUS) were detected were predominantly the cause of intellectual disability (ID), and in most cases, features of ASD as well. Using such an approach, variants in rarely described ASD-associated genes, such as *SIN3B*, *NR4A2*, and *GRIA1*, were detected. By expanding the analysis to include functionally similar NDD genes, variants in *KCNK9*, *GNE*, and other genes were identified. These would probably have been missed by classic genotype–phenotype analysis. Our study thus demonstrates that in patients with ASD, analysis of ultrarare or “single event” variants obtained using WES with the inclusion of functionally similar genes and reverse phenotyping obtained a higher diagnostic yield despite limited clinical data. The present study also demonstrates that most of the causative genes in our cohort were involved in the syndromic form of ASD and confirms their comorbidity with other developmental disorders.

## Introduction

Autism spectrum disorders (ASD) combine phenotypically heterogeneous groups that often co-occur with other neurodevelopmental disorders (NDDs) (Arteche-López et al., 2021). Clinically, two main features must be met for the diagnosis of ASD; the patient must present with difficulties in each of the three social communication subdomains and two of the four different restricted, repetitive sensory–motor behaviors in the past or present for both features (Lord et al., 2018). Variations in phenotype and their severity indicate that genetics and environmental factors are key players in the etiology of ASD (Rylaarsdam and Guemez-Gamboa, 2019). The size of genetic variants and mutation types vary from large chromosomal aberrations to single nucleotide variants (SNVs). They may also be rare or common risk variants (Balicza et al., 2019). Current guidelines for the detection of copy number variants (CNVs) with chromosomal microarray (CMA) in patients with NDDs, including ASD, as first-tier tests are still valid, although they were published over a decade ago (Miller et al., 2010).

Implementing next-generation sequencing (NGS) methods in the molecular diagnosis of NDDs has considerably increased the diagnostic yield (Lindstrand et al., 2019; Arteche-López et al., 2021). Whole exome sequencing (WES) enables the detection of rare or common small deletions, duplications, indels, synonymous, nonsense, splice, 3′-UTR, and missense or frameshift variants in the coding regions of the genome. The use of WES analysis in conjunction with CNV analysis enables a molecular diagnosis to be made in about 30% of patients with ASD and intellectual disability (ID) (Valentino et al., 2021). NGS methods have also enabled us to pinpoint genes implicated in ASD phenotypes (Bernier et al., 2014; Satterstrom et al., 2020). Trio-based WES analysis has proven to be successful in identifying rare inherited and *de novo* variants in genes involved in the etiology of ASD and novel ASD-associated genes (Satterstrom et al., 2020; Lintas et al., 2021). However, the inability to determine the origin of the suspected variant, reduced penetrance, broad clinical characterization of patients with ASD, or lack of clinical information aggravates the process of making proper genotype–phenotype correlations. Therefore, in these cases, it makes sense to reverse the diagnostic process by looking for ultrarare variants in the ASD-associated genes in the same group of patients and only then defining their clinical significance. This approach reduces the probability of an inaccurate analysis due to a highly heterogeneous phenotype or limited descriptions of patients by using simple algorithms. Following this approach, variants in rarely described ASD-associated genes, such as the *SIN3B*, *NR4A2*, and *GRIA1*, were detected, and a broad clinical phenotype combining ID and neurodevelopmental abnormalities, including ASD, was described in these patients (Geisheker et al., 2017; Lévy et al., 2018; Latypova et al., 2021). More importantly, the diagnostic yield was further increased using this type of analysis for potential causative variants in genes involved in other NDDs (the *KCNK9* and *GNE* genes). These genes were not linked to ASD according to ASD-associated genes listed in the Simons Foundation Autism Research Initiative (SFARI) database^1^ (Graham, Zadeh et al., 2016; Yang et al., 2020). Variants of unknown significance (VUS) also represent a challenging category for clinical interpretation. Therefore, for genes with VUS, variant protein function predictions and gene prioritizations were performed to identify causative genes outside of the ASD-associated pool of genes.

The present study further endorses the comorbidity of ASD and NDDs, indicating that genetic analysis should not be limited only to the wide range of ASD-associated genes. We also demonstrated how phenotype-driven analysis is not always the optimal approach, especially in cases with limited clinical data. The present study also demonstrates how the usage of the obtained genetic data in combination with simple step algorithms and bioinformatics could increase the yield of molecular diagnostics for patients with ASD.

## Materials and Methods

### Patient Enrollment

Patients with ASD from a large cohort of 439 Slovenian children with NDD were included (Krgovic et al., 2018). In this study, patients with ASD and their parents were re-invited to participate in the WES study. Written informed consent from their parents or legal guardians was obtained for all probands included in our cohort. This study is part of a larger study that was approved by the Commission of the Republic of Slovenia for Medical Ethics (KME No. 89/01/11). All experimental procedures were performed according to guidelines and regulations and abided by the tenets of the Declaration of Helsinki.

### Patients

A WES analysis was carried out on DNA samples from 147 pediatric patients with a suspected diagnosis of ASD. The patient group consisted of 110 boys (74.8%) and 37 girls (25.2%) with a mean age of 8.2 ± 0.935 years (±SD) at molecular diagnostic referral. The main referral diagnosis was suspected ASD, usually with coexistent ID, development delay (DD), language impairment, attention deficit hyperactivity disorder (ADHD), and other disorders. The test group included 10 pairs of siblings, of which 2 sets were twins, and 2 families had more than 2 affected children.

### Whole Exome Sequencing

Whole exome sequencing was performed on patients and, in some cases, their parents using DNA extracted from peripheral blood leukocytes with the QIAamp^®^ DNA Blood Midi Kit (QIAGEN, Hilden, Germany). WES and library construction were performed by Novogene (Novogene Company Limited, Cambridge, United Kingdom) using the Agilent SureSelect Human All Exon Kit (Agilent Technologies, Santa Clara, CA, United States). The DNA libraries were then sequenced on an Illumina HiSeq 4000 platform (150-bp paired-end reads) (Illumina, San Diego, CA, United States) with an average depth coverage of 100× with an average 12 Gb output per sample.

### Analysis Algorithm

**FIGURE 1:**
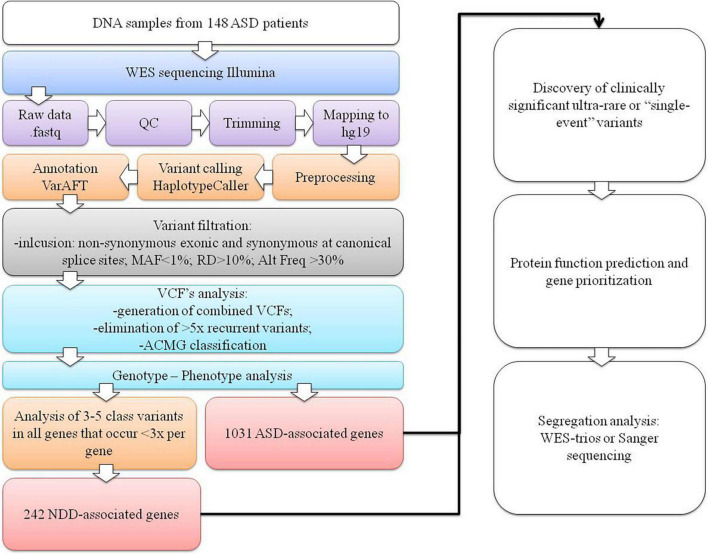
The WES data variant analysis step-by-step workflow used in our cohort. The first section represents the NGS pipeline for generating VCF files. In the second section, variant filtering, identifying ultrarare or “single event” variants process, and data interpretation are presented.

Raw fastq reads were first assessed for quality control using FastQC v0.11.9 software (Wingett and Andrews, 2018). The Trimmomatic tool v0.39 was used to trim technical sequences and adapters from raw sequences (Bolger et al., 2014). The Burrows-Wheeler Aligner BWA v0.7.12-r1039 with the MEM algorithm was used for mapping the raw reads to the human reference genome (GRCh37/hg19) (Li and Durbin, 2010). PCR duplicates’ marking and mate information fixation were performed using PicardTools v2.20.1. Base quality scores were additionally recalibrated using the genome analysis toolkit (GATK) v4.0 (McKenna et al., 2010; DePristo et al., 2011; Van der Auwera et al., 2013). Variant calling was performed using the HaplotypeCaller algorithm in genomic mode with joint calling (Poplin et al., 2018). Variant quality score recalibration using a GATK-implemented adaptive error model, a machine learning method with known genomic sites, was also used to establish a method for enabling variant filtering based on the fine balance between sensitivity and specificity for true or false genetic variants. Normalization with left trimming was also performed using GATK to obtain the final unfiltered VCF files. Variant annotation was performed using the VarAFT tool v2.17-2 (Desvignes et al., 2018). Machine learning algorithms using conservation profiles, such as SIFT and PolyPhen-2 conservation profiles, were used for the prediction of consequences of missense mutations (Kumar et al., 2009; Adzhubei et al., 2010, 2013). Subsequently, stepwise variant filtering was performed for variants with a read-depth coverage of >10× and an altered variant frequency of at least 30%. Variants with a frequency of more than 1% in the population variant databases [1,000 genome projects, Exome Aggregation Consortium (ExAC), and Exome Variant Server (EVS)] were excluded. Intergenic or 3′-UTR/5′-UTR untranslated region variants and non-splice–related intronic and synonymous variants were also filtered out, except for those located at canonical splice sites (Figure 1).

### Variant Filtering and Classification

Filtered VCF files for all 147 patients were combined, generating a catalog of 39,668 variants (on average, 268 variants per patient), which were sorted by their canonical transcript and genomic position. All the recurrent variants that were present more than five times were eliminated as they were presumed to be sequence artifacts. We then obtained only ultrarare variants or “single event” variants that occurred only once per gene. Variants were then classified based on standards and guidelines of the American College of Medical Genetics and Genomics (ACMG) as pathogenic (class 5), likely pathogenic (class 4), variants of uncertain clinical significance (VUS) (class 3), likely benign (class 2), and benign (class 1) using the Franklin^2^ tool (Richards et al., 2015). For all variants classified as possible causative (classes 3–5) in 1,031 genes listed in the SFARI database (SFARI-Gene_genes_01-11-2022release_03-23-2022export) (Supplementary Table 1), genotype–phenotype analysis was performed based on the clinical description of patients and a literature search. If parents’ samples were available, segregation analysis with WES-trio for VUS or Sanger sequencing for likely pathogenic or pathogenic variants was carried out.

The analysis was further expanded for all class 3–5 variants in all genes, with the limitation that no more than three variants emerged per gene. This facilitated detection of the rare or “single event” variants in the gene that was more likely to be causal and reduced the scope of the analysis. For these genes, a subsequent extensive literature search was performed to determine whether the gene could be associated with NDDs, resulting in the inclusion of an additional 242 NDD-associated genes (Supplementary Table 1) in the genotype–phenotype analysis. The diagram of our analysis is presented in Figure 1.

### Segregation Analysis

Segregation of the potential variant was performed in 32 trio-based WES analyses, 10 family WES analyses (siblings and parents), and Sanger sequencing in 14 families when pathogenic/likely pathogenic variants in ASD-associated genes were detected. All variants detected by WES analysis were then confirmed by Sanger sequencing using the Beckman Coulter CEQ 8000 Genetic Analysis System (Beckman Coulter Inc., Indianapolis, IN, United States).

### Protein Function Predictions and Gene Prioritizations

The STRING^3^ tool was used for generating functionally similar genes based on genomic and proteomic data for the detected pathogenic, likely pathogenic, and VUS variants (Table 2; Szklarczyk et al., 2019). The genes were clustered with *k*-means clustering into four groups to identify possible correlations in the same biological pathways and/or involvement in specific phenotypes (diseases).

The same set of genes was also used in enrichment and Gene Ontology (GO) analysis, employing the g:Profiler^4^ tool to determine which Human Phenotype Ontology (HP) terms are most frequently linked with the analyzed genes, particularly for genes with VUS variants that are not listed in the SFARI database (Raudvere et al., 2019).

## Results

### Whole Exome Sequencing and Segregation Analysis

With WES analysis focusing on ultrarare variants in 1,031 ASD-associated genes, we were able to identify pathogenic or likely pathogenic variants, 14 of which were *de novo*, in 20.4% (N 30/147) of patients. In two patients, two variants were identified. One of these patients was presumed to be compound heterozygous with *de novo* and maternally inherited variants, while the other patient had a likely pathogenic and a VUS variant in two different genes. One patient has a maternally inherited variant that was also present in the younger brother with phenotype consistency. In one patient, a variant was excluded in the mother but not in the father. Segregation was not tested in 13 patients as parental samples were not available for further analysis. One of these 13 patients was the carrier of both the likely pathogenic and VUS variants in 2 different genes. The diagnostic yield was further increased by 2.7% (N 4/147) by adding the analysis of ultrarare variants detected in an additional 242 NDDs genes not listed in the SFARI database (Supplementary Table 1). Of these genes, one variant was *de novo*, one was maternally inherited, one variant was not from the mother, and segregation was not analyzed in one variant. The overall diagnostic yield was estimated to be 23.1% (N 34/147). VUS variants were detected in 10.2% (N 15/147) of patients, of which 4.1% (N 6/147) were detected by adding additional NDDs gene analysis. In 11 patients with VUS variants in all genes together, segregation was not tested, 1 was maternally and 2 were paternally inherited. In one patient, inheritance was excluded from the mother but not from the father, and one had a *de novo* variant. Our results are summarized in Table 1. Patient characteristics and identified pathogenic, likely pathogenic, and VUS variants are listed in Table 2.

**TABLE 1:** Diagnostic yield obtained by WES in 147 patients with suspected ASD.

	1,031 ASD-associated genes		242 NDD-associated genes	
	LP/P	VUS	LP/P	VUS
	
No. of ASD patients	30/147 (20.4%)	9/147 (6.1%)	4/147 (2.7%)	(6/147) 4.1%
No. of patients with more than one variant	3		1	

No. of variants detected	31	12	4	6

**Inheritance**				
*De novo*	15*	1	1	/
Maternal	2*	/	1	1
Paternal	/	/	/	2
Not tested	13	10	1	3
Excluded from one parent	1	1	1	/

*One de novo and maternally inherited variant found in the same gene in a presumed compound heterozygous state.LP, likely pathogenic variant; P, pathogenic variant; VUS, variant of unknown significance.

**TABLE 2:** Description of patients clinical characteristics for molecular diagnostic referral and identified as pathogenic, likely pathogenic, and VUS variants in the genes.

Patient ID	Sex	Age at referral (years)	Clinical indication for referral	Gene	HGVS nomenclature	Protein change	Zygosity/origin	ACMG classification	OMIM and/or literature	DECIPHER^1^ ID/HGDM^2^ identifier
025244	F	14	Asperger syndrome, ADHD, OCD, psychiatric problems	*ABCA13*	NM_152701.5:c.8953dup	p.(Gln2985ProfsTer4)	het/NA	LP	Chen et al., 2017	480974/NA
033532	M	4	ASD, ID	*ACTL6B*	NM_016188.5:c.694C > A	p.(Pro232Thr)	het/NA	VUS	Bell et al., 2019	480976/NA
021055	M	10	ASD, ID	*ADNP*	NM_015339.4:c.2188C > T	p.(Arg730Ter)	het/*dn*	P	Arnett et al., 2018	480982/CM166963
027819	M	15	Suspected ASD, mild ID, ADHD, aggressive behavior, absent speech, bilateral hearing impairment, long face with prominent ears, micrognathia	*ADNP*	NM_015339.4:c.3071_3072del	p.(Glu1024AlafsTer7)	het/NA	LP	Arnett et al., 2018	480984/NA
020213	F	14	ASD, gross DD, ID, microcephaly, absent speech, low-set ears	*ADNP*	NM_015339.4:c.2496_2499del	p.(Asn832LysfsTer81)	het/*dn*	P	Arnett et al., 2018	480985/CD144180
035442	F	19	ASD, ID, aggressive, speech delay	*CAMK2B*	NM_001220.5:c.328G > A	p.(Glu110Lys)	het/NA	LP	Rizzi et al., 2020	480986/CM1716240
045750	F	2	ASD, DD, microcephaly	*CHD8*	NM_001170629.2:c.7181A > G	p.(Lys2394Arg)	het/NA	VUS	Du et al., 2018	480987/NA
041619	M	5	ASD, DD, speech delay, ADHD	*CSNK1E*	NM_001894.4:c.188-1G > T	p.?	het/NA	LP	Chen et al., 2019	480989/NA
046526	M	4	ASD, ID, ADHD	CYFIP2*	NM_001037333.3:c.40G > A	p.(Val14Met)	het/NA	VUS	Begemann et al., 2021	480992/NA
040054	M	15	ASD, ID, disproportionate tall stature, arachnodactyly, downward slanting palpebral fissures, EEG abnormality, poor fine motor coordination, delayed speech and language development, OCD, dental crowding, flat face, kyphoscoliosis, joint hypermobility, increased arm span, abnormality of the sternum	*DNMT3A*	NM_175629.2:c.1969G > A	p.(Val657Met)	het/not inh mat and not present in healthy sister	VUS	Tatton-Brown et al., 2018	480994/NA
025324	M	8	ASD, ADHD, mild ID, speech delay	*DLG2*	NM_001142699.1:c.285A > G	p.(Gln95Gln)^a^	het/*dn*	VUS	Ruzzo et al., 2019	480996/NA
016930	F	7	ASD, ID/DD, microcephaly, hypotonia, epilepsy	*DYRK1A*	NM_001396.4:c.1316del	p.(Ala439ValfsTer12)	het/*dn*	P	Earl et al., 2017	480999/NA
047559	M	6	ASD, DD, speech delay, brachycephaly, twin brothers	FGFR2*	NM_022970.3:c.1069G > T	p.(Val357Phe)	het/pat	VUS	Azoury et al., 2017; Szczurkowska et al., 2018	481000/NA
047560	M	6		FGFR2*	NM_022970.3:c.1069G > T	p.(Val357Phe)	het/pat	VUS		481002/NA
024441	M	7	ASD, ID, psychosis	*FOXP2*	NM_148898.4:c.1674G > A	p.(Trp558Ter)	het/NA	LP	Trelles et al., 2021	481004/NA
039934	F	4	ASD, absent speech, poor eye contact, poor social contact	*FBXO11*	NM_001190274.1: c.1112G > T	p.(Ser371Ile)	het/NA	VUS	Jansen et al., 2019; Gregor et al., 2022	481005/NA/NA
				*HECW2*	NM_020760.3:c.4436G > A	p.(Arg1479Gln)	het/NA	LP	Ullman et al., 2018	
041707	M	6	ASD, DD, poor/absent speech	*HECW2*	NM_020760.3:c.4294-1G > T	p.?	het/NA	LP	Ullman et al., 2018	481007/NA
035247	M	5	ASD, DD	GNE*	NM_001128227.2: c.1287dup	p.(Asp430ArgfsTer3)	het/NA	LP	Yang et al., 2020	481008/NA
045124	F	5	ASD, ID, global DD, delayed speech and language development	*GRIA1*	NM_001258021.1: c.1526G > A	p.(Arg509Gln)	het/*dn*	LP	Geisheker et al., 2017	481010/NA
045232	M	10	Suspected ASD, ADHD, mild ID *^b^*Younger brother with similar problems – not tested for variant in the *GRIA4* gene	GRIA4*	NM_000829.4:c.627T > G	p.(Phe209Leu)	het/NA	VUS	Martin et al., 2017	481011/NA
046940	M	4	ASD	GRIA4*	NM_000829.4:c.1064A > C	p.(Gln355Pro)	het/NA	VUS	Martin et al., 2017	481013/NA
018325	M	11	ASD, DD	*GRIN2A*	NM_000833.4:c.982G > T	p.(Glu328Ter)	het/NA	LP	Strehlow et al., 2019	481017/NA
016906	M	6	ASD, ID/DD, ADHD	*GRIN2A*	NM_000833.5:c.4204C > T NM_000833.5:c.2329C > G	p.(Arg1402Trp) p.(Leu777Val)	het/NA het/NA	VUS VUS	Strehlow et al., 2019	481018/NA/NA
027448	M	19	ASD, generalized-onset seizure, developmental regression, feeding difficulties, absent speech, poor fine motor coordination, progressive inability to walk, thoracolumbar scoliosis	*IQSEC2*	NM_001111125.3:c.2563C > T	p.(Arg855Ter)	hemy/*dn*	P	Lopergolo et al., 2021	481019/CM129316
019020	M	9	Autistic behavior, ID, long face, cleft palate, strabismus *^b^*Younger brother with ADHD but without *KCNK9* mutation	KCNK9*	NM_001282534.2:c.392G > A	p.(Arg131His)	het/*dn*	LP	Graham, Zadeh et al., 2016	481021/NA
019450	M	13	ASD, epilepsy	*KCNQ2*	NM_172107.4:c.1997C > T	p.(Pro666Leu)	het/NA	VUS	van der Werf et al., 2020	481022/NA
037249	M	7	Asperger syndrome, ADHD, epilepsy	*KCNQ5*	NM_001160133.2:c.911G > C	p.(Trp304Ser)	het/NA	VUS	Lehman et al., 2017	481023/NA
033739	M	5	ASD, DD	*KDM5A*	NM_001042603.2:c.953A > G	p.(Tyr318Cys)	het/NA	VUS	El Hayek et al., 2020	481024/NA
040353	M	12	Autistic behavior, delayed speech and language development, stereotypical hand wringing, poor eye contact	*KDM6B*	NM_001080424.2:c.3196_3199dup	p.(Ala1067ValfsTer31)	het/*dn*	P	Stolerman et al., 2019	481025/NA
047960	M	5	Autistic behavior, ADHD, poor motor coordination, speech delay	*KMT2C*	NM_170606.3:c.11586_11587del	p.(Pro3863SerfsTer18)	het/NA	LP	Koemans et al., 2017	481026/NA
029613	M	3	Autistic behavior, abnormal aggressive, impulsive or violent behavior, neurodevelopmental delay	*LZTR1*	NM_006767.4:c.451G > A	p.(Asp151Asn)	het/*dn*	LP	Johnston et al., 2018	481035/NA/NA
				*LZTR1*	NM_006767.4:c.1672C > T	p.(Gln558Ter)	het/mat	LP		
037023	M	7	Autistic behavior, ID, global DD, language impairment, hypotonia, pain tolerance, sleep disturbance	MAGT1*	NM_032121.5:c.527C > T	p.(Ala176Val)	hemy/mat	VUS	Blommaert et al., 2019	481036/NA
033763	F	12	Autistic behavior, ID/DD, delayed gross motor development, abnormal emotion/affect behavior, childhood stereotypy	*MECP2*	NM_001110792.2:c.961C > T	p.(Arg321Trp)	het/*dn*	P	Liyanage and Rastegar, 2014	481037/NA
018653	F	12	ASD, ID	*NR4A2*	NM_006186.4:c.571C > T	p.(Gln191Ter)	het/*dn*	P	Lévy et al., 2018; Guo et al., 2019	481038/NA
027329	M	5	ASD, ID, delayed speech and language development, high pain tolerance ^b^Younger brother: ASD, DD, absent speech, stereotypic movement – *NRXN3* mutation carrier ^b^Mother with mild ID, delayed speech and language development in childhood	*NRXN3*	NM_004796.6:c.526C > T	p.(Arg176Ter)	het/mat	LP	Wang et al., 2018	481039/NA
022629	M	11	ASD, ID	*PPP2R5D*	NM_006245.4:c.592G > A	p.(Glu198Lys)	het/*dn*	P	Biswas et al., 2020	481040/CM153575
038924	F	11	ASD, ID	*PPP2R5D*	NM_006245.4:c.758G > A	p.(Arg253Gln)	het/NA	LP	Biswas et al., 2020	481041/NA
035820	F	12	ASD, ADHD, ID/DD, motor delay, delayed speech and language development, muscular hypotonia	*RAI1*	NM_030665.4:c.1854del	p.(Ile618MetfsTer201)	het/*dn*	P	Abad et al., 2018	481042/NA
036247	M	4	Autistic behavior, DD, epilepsy	*RPS6KA3*	NM_004586.3:c.1631A > G	p.(Asn544Ser)	hemy/NA	LP	Field et al., 2006	481046/NA
018960	F	12	Autistic behavior, global DD, absent speech	*SCN8A*	NM_014191.4:c.57del	p.(Glu20SerfsTer70)	het/not inh mat	LP	Butler et al., 2017	481048/NA
027720	M	3	ASD, DD	*SIN3B*	NM_015260.4:c.1843G > A	p.(Asp615Asn)	het/*dn*	LP	Latypova et al., 2021	481049/NA
034742	F	13	Autistic behavior, cognitive impairment, short attention span, motor delay, visual impairment, delayed speech and language development, poor fine motor coordination, prominent nasal tip, microcephaly, recurrent infections, lactose intolerance	SLC2A1*	NM_006516.4:c.667C > T	p.(Arg223Trp)	het/not inh mat	P	López-Rivera et al., 2020; Mir et al., 2022	481051/CM101705
023509	F	7	ASD	*SOS2*	NM_006939.4:c.791C > A	p.(Thr264Lys)	het/*dn*	P	Yamamoto et al., 2015	481052/CM1511144
019146	F	6	ASD, ID	*STAG1*	NM_005862.3:c.2672A > C	p.(Lys891Thr)	het/NA	VUS	Lehalle et al., 2017	481053/NA
022940	M	4	ASD, twin brothers	*TBL1XR1*	NM_024665.7:c.303_304del	p.(Asp101GlufsTer43)	het/NA	LP	Quan et al., 2020	481054/NA
022941	M	4		*TBL1XR1*	NM_024665.7:c.303_304del	p.(Asp101GlufsTer43)	het/NA	LP		481055/NA
040139	M	5	ASD, DD, neonatal hypertonia, poor fine motor coordination, delayed speech and language development, umbilical hernia, atopic dermatitis, dental hypoplasia, abnormal palate morphology	*TBL1XR1*	NM_024665.7:c.1183T > C	p.(Tyr395His)	het/*dn*	P	Quan et al., 2020	481056/NA
040110	M	14	ASD, ADHD, hearing impairment, obesity, panhypopituitarism	*TM4SF20*	NM_024795.4:c.184-2A > T	p.?	het/not inh mat	VUS	Andres et al., 2021	481057/CS1826517/NA
				KRAS*	NM_033360.4:c.401C > G	p.(Ala134Gly)	het/mat	LP	Roberts, 2001; Struja and Capraro, 2021	
011139	M	7	Autistic behavior, ADHD, ID, motor delay, delayed speech and language development	*TRIP12*	NM_004238.3:c.4813dup	p.(Val1605GlyfsTer17)	het/*dn*	P	Louie et al., 2020	481059/NA

*^1^https://www.deciphergenomics.org/.*

*^2^http://www.hgmd.cf.ac.uk/ac/index.php.*

*^a^Synonymous variant with potential altered splice effect.*

*^b^Observed positive family history.*

^*^ NDD-associated genes not listed in the SFARI gene list.

*HGVS, Sequence Variant Nomenclature; het, heterozygous; hemy, hemizygous; NA, not available; dn, de novo; mat, maternal; pat, paternal; not inh mat, excluded in the mother; P, pathogenic; LP, likely pathogenic; VUS, variant of unknown significance.*

**FIGURE 2:**
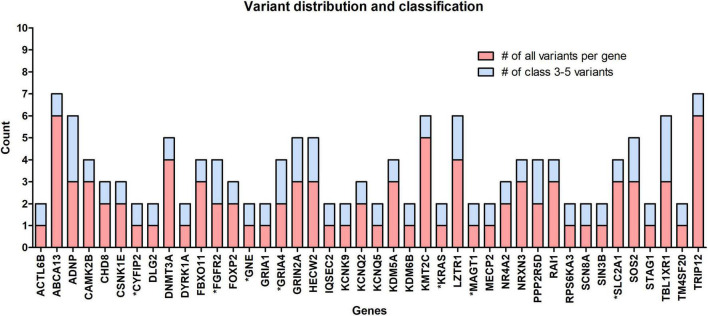
The occurrence of all rare variants per gene (red) and the number of those classified as clinically significant variants (blue). In 18 out of 42 identified genes, 1 variant per gene occurred, the so-called “single event” variant. Genes marked with an asterisk (*) belong to the group of NDD-associated genes.

The occurrence of variants per gene for clinically significant variants analyzed in this cohort is presented in Figure 2. From the chart in the figure, it can be seen that in 18 out of 42 genes, the variant occurred as a “single event” (meaning we detected 1 variant per gene in all patients combined). Benign and likely benign variants were detected in the *ABCA13*, *DNMT3A*, *KMT2C*, and *TRIP12* genes. In the other genes, only ACMG class 3–5 variants were found.

### Protein Function Predictions and Gene Prioritizations

Functional enrichment analysis of 42 genes showed that they were predominantly involved in the development of the nervous system with a *p*-value of 4.16e−06 (N 22/42). Genes can be ranked into three major groups according to their molecular function. The first group consists of chromatin-binding proteins (GO:0003682): ACTL6B, ADNP, CHD8, DNMT3A, KDM5A, KDM6B, MECP2, SIN3B, and STAG1. The second group consists of proteins with gate channel activity: GRIA1, GRIA4, GRIN2A, KCNK9, KCNQ2, KCNQ5, and SCN8A. The third group consists of proteins that share transcriptional regulatory function (GO:0140110) with ADNP, ACTL6B, KDM5A, MECP2, and SIN3B proteins, and they include DYRK1A, FOXP2, KMT2C, LZTR1, NR4A2, RAI1, and TBL1XR1. Other proteins are involved in processes, such as transmembrane transporter activity (GO:0022857) (ABCA13, MAGT1, and SLC2A1) and regulation of molecular functions (GO:0098772) (IQSEC2, PPP2R5D, RPS6KA3, and SOS2).

**FIGURE 3:**
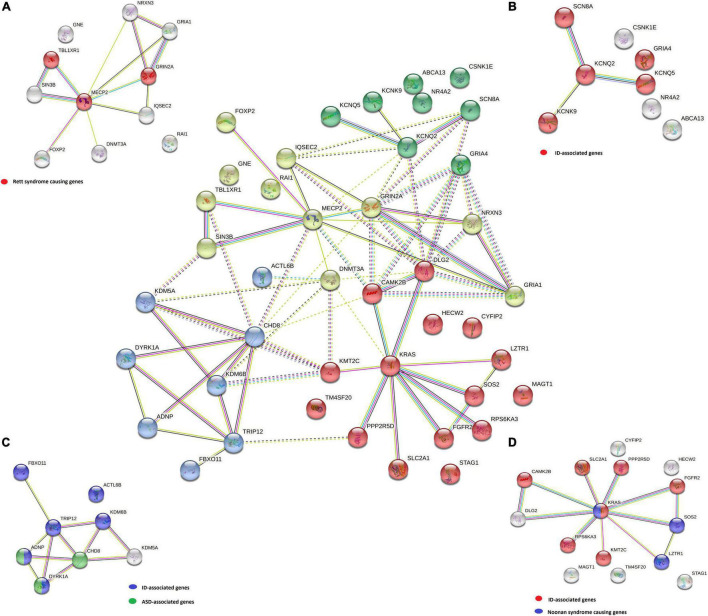
A STRING *k*-means clustering classified the genes into four groups: yellow **(A)**, green **(B)**, blue **(C)**, and red **(D)** by possible correlation in the same biological pathways and/or involvement in specific phenotypes (diseases). For each group, specific disease-gene associations are marked on the isolated figure forms **(A–D)**.

Disease-gene associations showed that 28 out of 42 genes analyzed were involved in mental health diseases (*p*-value of 4.97e−27), of which 22 genes were also linked to ID (*p*-value of 8.58e−23). Only four of these genes (*ADNP*, *CHD8*, *DYRK1A*, and *MECP2*) were linked to ASD (*p*-value of 7.9e−4). Clustering 42 genes using the STRING tool generated 4 gene clusters that were colored red (15 genes), yellow (11 genes), blue (8 genes), and green (8 genes). The largest red cluster consisted of three Noonan-causing genes *KRAS*, *LZTR1*, and *SOS2* (Johnston et al., 2018). The *KRAS* gene interacted with other genes in this node, as well as the shared ID in disease-gene association with six other genes (colored red in the isolated figure form). The yellow-colored node encompassed the three Rett syndrome-causing genes *MECP2*, *GRIN2A*, and *TBL1XR1* (colored red in Figure 3A), but mutations in other genes in this node were also involved in a Rett-like phenotype (Wang et al., 2019). The blue-colored node consists of the ASD-associated genes *ADNP*, *CHD8*, and *DYRK1A* (colored green in Figure 3C), but the majority of genes in this node are predominantly linked to ID (colored blue in Figure 3C). Functional enrichment analysis in this node also showed that five of the eight proteins (ACTL6B, ANDP, CHD8, KDM5A, and KDM6B) were chromatin binding (GO:0003682) (*p*-value of 3.6e−3). The green-colored node consists of eight genes, six of which code for proteins involved in transmembrane transporter activity (GO:0022857) (*p*-value of 3.6e−3) and were also linked to ID (colored red in the isolated figure form) with the exception of the *ABCA13* gene.

All four gene groups are presented in the center of Figure 3. Disease-gene associations are labeled for each group separately in Figures 3A–D.

**FIGURE 4:**
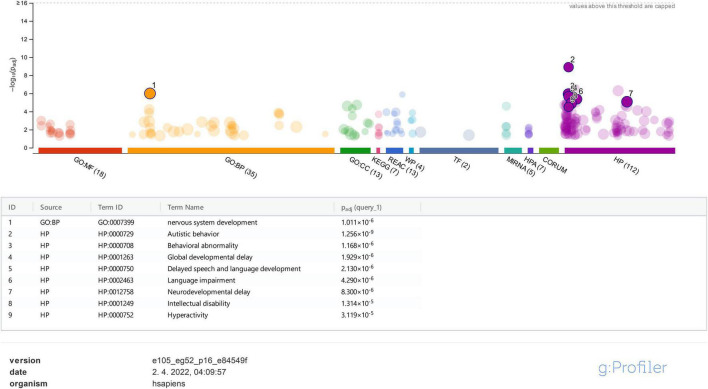
g:Profiler analysis for the studied genes based on GO is presented for molecular function (MF), biological process (BP), cellular component (CC), biological pathways (KEGG, Reactome, and WikiPathways), regulatory motifs in DNA (TF and MIRNA), protein databases (HPA and CORUM), and Human Phenotype Ontology (HP). Only data for BP and HP were analyzed and represented in the figure.

On the contrary, using the g:Profiler tool for the same set of genes, it was clearly shown that ASD under HP:0000729 (purple bubble 2 in Figure 4) is the main feature (*p*-value of 2.457e−10) linked to the analyzed set of genes. Features, such as delayed speech and language, language impairment, global DD, neurodevelopmental delay, ID, and hyperactivity, and also the features observed in our patients, were significantly found less frequently than ASD (purple bubbles 3–8 in Figure 4). As observed in the STRING analysis, the GO biological process analysis showed that the analyzed genes were predominantly involved in the development of the nervous system (GO:0007399) (*p*-value of 1.878 × e−6). All the data from the g:Profiler analysis are available online.

## Discussion

The introduction of WES in the genetic diagnosis of ASD has significantly improved our knowledge of its etiology at the molecular level. The phenotype-driven analysis is often a limitation in the discovery of new disease-associated genes since the clinical presentation of patients with ASD is a key factor in genetic testing and data interpretation (Arteche-López et al., 2021; Lintas et al., 2021). The process of establishing an accurate diagnosis is further hindered by the lack of segregation analysis, reduced penetrance, limited data availability in the literature, as well as non-specific and limited clinical descriptions of patients. Therefore, reverse phenotyping and seeking the ultrarare variants in genes that are not only limited to ASD and then performing functional studies to obtain datasets should result in a higher diagnostic yield (Lintas et al., 2021).

In our study, a WES analysis was performed in 147 pediatric patients with suspected ASD. Genotype–phenotype analysis of 1,031 ASD-associated genes enabled the detection of pathogenic or likely pathogenic variants in 20.4% (N 30/147) of patients, of which almost half (N 14/30) were shown to be *de novo* in subsequent segregation analysis. The high incidence of *de novo* variants was expected in our study since it is estimated that *de novo* events contribute to autism in 30–39% of all patients with ASD (Yoon et al., 2021). In addition, one had a maternally inherited variant, and one patient was presumed to be a compound heterozygote, harboring one maternally inherited and one *de novo* variant. Adding 242 NDD-associated genes (Supplementary Table 1) to the genotype–phenotype analysis resulted in the detection of pathogenic/likely pathogenic variants in four more patients and increased the diagnostic yield to 23.1% (N 34/147). A VUS variant was detected in 10.2% (N 15/147) of patients. All the variants and patient clinical characteristics are listed in Tables 1, 2.

Protein function predictions and gene prioritization analyses were performed for genes identified in our cohort to establish possible correlations between already known ASD-associated genes and those not listed in the SFARI database. The disease-gene associations analysis (Figure 1) shows that the majority of detected variants are associated with an ID phenotype, and thus we presumed that our patients mostly have a syndromic ASD since autistic behavior (HP:0000729) is strongly associated with all of the analyzed genes (Figure 4). According to the literature, ASD is comorbidity with ID in 32% of patients (Miot et al., 2019). Our disease-gene associations showed that 22 out of 42 genes analyzed were also linked to ID (*p*-value of 8.58e−23), whereas only 4 genes (*ADNP*, *CHD8*, *DYRK1A*, and *MECP2*) were linked to ASD (*p*-value of 7.9e−4). As expected, functional enrichment analysis confirmed that genes involved in the etiology of ASD were predominantly involved in the development of the nervous system, and mutations in these genes, in our cases, altered their transcriptional regulatory functions or transmembrane transporter activity, which are known molecular mechanisms in the etiology of ASD (Rylaarsdam and Guemez-Gamboa, 2019).

Functional enrichment analysis for genes not listed in the SFARI database also demonstrated their association with the already known ASD-associated genes. For example, based on their functional similarity, the *KRAS*, *SLC2A1*, and *FGFR2* genes were clustered in the red node. The *KRAS* gene, along with *LZTR1* and *SOS2*, is one of the three Noonan syndrome-causing genes in this group. They cause dysregulation of the RAS-MaPK signaling pathway (Tartaglia et al., 2011) and are also linked to ID (Figure 3D). The maternally inherited pathogenic variant in the *KRAS* gene explains the panhypopituitarism observed in our patient (Patient 040110) (Struja and Capraro, 2021). Although the *KRAS* gene is not linked to ASD, ASD was observed in Patient 040110, presumed compound heterozygous variants in *LZTR1* in Patient 029623, and a *de novo* variant in *SOS2* in Patient 023509, are a probably causative in all three patients since ADHD and behavioral problems are reported in the Noonan syndrome phenotype (Roberts, 2001). For Patient 029623, additional analyses should be performed to establish whether the variants are truly biallelic since compound heterozygosity was presumed based only on the genotype–phenotype correlation. Patient 040110 is also a carrier of a VUS variant in the *TM4SF20* gene with a predicted acceptor loss score of Δ0.99 using the SpliceAI tool,^5^ associated with language impairment (Andres et al., 2021). However, this feature was not reported in our patient (Table 2). This can be explained by the reduced penetrance observed for *TM4SF20* truncation carriers (Wiszniewski et al., 2013). Our analysis showed that the *KRAS* gene is functionally linked to the *SLC2A1* gene, which is also not reported as an ASD-associated gene. The abovementioned connection stems from tumor genetics since glucose deprivation has been shown to contribute to the development of *KRAS* pathway mutations in tumor cells (Yun et al., 2009). A presumed *de novo* pathogenic mutation in the *SLC2A1* gene was detected in a 13-year-old girl (Patient 034742) with a severe phenotype, which reverse phenotyping has shown, is a cause of the GLUT1 deficiency syndrome (López-Rivera et al., 2020; Mir et al., 2022). Another gene, not reported to be associated with ASD, but which can be linked to *KRAS*, is the *FGFR2* gene since they are both involved in the RTK/Ras/MAPK signaling pathway (Das et al., 2014). The paternally inherited VUS variant was detected by family-WES analyses in twin brothers with ASD, DD, speech delay, and brachycephaly. Mutations in the *FGFR2* gene with incomplete penetrance have been reported to cause a syndromic form of craniosynostosis with ID/DD (Azoury et al., 2017). To the best of our knowledge, no clear association of this gene with ASD has been established, although a functional study in mice has shown that dysregulation of the *Fgfr2* gene leads to impaired core behavior related to ASD (Szczurkowska et al., 2018). For all three abovementioned genes, the g:Profiler tool showed involvement with high significance in behavioral abnormalities (HP:0000708), DD (HP:0001263), ID (HP:0001249), and also in autistic behavior (HP:0000729) for the *SLC2A1* gene (Figure 4 and data available online).

Similar correlations can be made for other non-SFARI genes in the other three clusters mentioned above. Two such genes are the *KCNK9* and *GRIA4* genes in the green cluster (Figure 3B). The pathogenic variant in the *KCNK9* gene was identified as a “single event” variant in NDD-associated genes (Figure 2). Reverse phenotyping revealed that Patient 019020 has a clinical feature of the *KCNK9* imprinting syndrome (Graham, Zadeh et al., 2016). Segregation analysis revealed a *de novo* variant, presumably on the maternal allele.

Two different VUS variants were detected in the *GRIA4* gene in two male patients (Patients 045232 and 046940) who share ASD as a common clinical feature. Segregation analysis was not performed in either case. Although the *GRIA4* gene is not listed in the SFARI database, *de novo* heterozygous pathogenic variants in the *GRIA4* gene have already been reported in patients with an ASD-like phenotype (Martin et al., 2017). The *KCNK9* and *GRIA4* genes were also clustered together with *KCNQ2*, *KCNQ5*, and *SCN8A* (Figure 3B). Five of eight genes in this cluster share gate channel activity (GO:0022836) and are involved in the ASD/ID/DD phenotype according to g:Profiler analysis (data available online).

Additional gene correlations were also identified by gene clustering. In the yellow node, according to WikiPathways data (Martens et al., 2021), there are three Rett syndrome-causing genes (Figure 3A). Genes, such as *IQSEC2* and even *KCNQ2* from the green node, are linked to these genes since both genes are involved in the Rett-like syndrome phenotype (Wang et al., 2019).

Despite an attempt to establish a true protein function prediction analysis for some genes in our cohort, no connection could be made. Their role in a patient’s phenotype was set by focusing on “single event” variants. In almost half of the cases (18 out of 42 genes), the variant occurred as a “single event.” This means that we detected one variant per gene in all patients combined (Figure 2), which confirms that ultrarare variants are a key factor in the etiology of ASD, and new genes will emerge by adding new patient cohorts. Among non-SFARI listed genes, variants were detected in the *CYFIP2*, *GNE*, and *MAGT1* genes. Using the g:Profiler tool, all three genes were found to be linked to NDD features (data available online), while the same three genes have been described in patients with an ASD-associated phenotype in the literature in recent years (Blommaert et al., 2019; Yang et al., 2020; Begemann et al., 2021). In the future, functional studies, variant segregation, and better genotype–phenotype analysis should be performed for this category of genes to establish their true roles in a patient’s phenotype.

These same studies should be performed for Patient 039934, a female with variants in both the *FBXO11* and *HECW2* genes, which are both associated with a complex NDD phenotype that includes autistic features, ID, and abnormalities in language development, features also present in our proband. A splice variant in the *HECW2* gene with a predicted acceptor loss score Δ of 1.00 using the SpliceAI tool was detected in Patient 041707 with a similar phenotype consisting of ASD, DD, and poor/absent speech. Since segregation analysis was not performed in either of these patients, the true significance of the variants for the patients’ phenotype is not clear. For both genes, *de novo* variants in the genes were reported as causative, hence variant segregation and better genotype–phenotype analysis are essential (Ullman et al., 2018; Jansen et al., 2019). Therefore, despite both variants in the *HECW2* gene being classified as likely pathogenic, at present, they should be treated as VUS.

By defining ultrarare and “single event” variants in the ASD-associated genes, variants in rarely reported patients with ASD in the literature were identified.

A pathogenic variant, later established as *de novo* after reverse phenotyping and segregation, was detected in a 12-year-old girl (Patient 018653) with ASD and ID. The *NR4A2* gene encodes nuclear receptor 4A subfamily (Nr4a) of transcription factors involved in hippocampal synaptic plasticity and cognitive functions (Català-Solsona et al., 2021). *NR4A2* haploinsufficiency caused by deletions or a frameshift mutation in this gene and associated with ID and ASD has been reported in the literature (Lévy et al., 2018; Guo et al., 2019). To the best of our knowledge, our patient has the first stop-gain mutation reported in the literature that confirms a *de novo* loss-of-function (LoF) mutation involved in the etiology of ASD. A different variant (Figure 2), also found in the *NR4A2* gene in another patient, was marked as not causative since it was also present in the mother who had no health problems.

In a 5-year-old girl with ASD, ID/DD, and delayed speech and language development (Patient 045124), a “single event” variant is a likely pathogenic *de novo* variant in the *GRIA1* gene. The GRIA1 gene encoding the α-amino-3-hydroxy-5-methyl-4-isoxazolepropionic acid (AMPA) receptor subunit GLUA1 has been reported in wide neurological and psychiatric disorders, including schizophrenia (Bygrave et al., 2019). In a large study of 17,688 patients with NDDs, 21 patients were carriers of a mutation causing a 636 amino acid change from alanine to threonine in the GRIA1 protein. These patients showed evidence of specific learning disabilities and autism (Geisheker et al., 2017). Hence, our patient represents a new mutational and phenotypic spectrum of patients with *GRIA1* gene haploinsufficiency.

A missense variant in the *SIN3B* gene was also found as a “single event” variant in Patient 023509 with ASD and DD. This variant was classified as a VUS according to ACMG guidelines, but WES-trio showed that the variant occurred *de novo*. The *SIN3B* gene encodes a transcription corepressor which has an important role in histone deacetylation and transcriptional repression (Latypova et al., 2021). An animal model showed that *Sin3b* knockout mice and zebrafish *sin3b* mutants express skeletal and growth defects (David et al., 2008; Moravec et al., 2017). *Sin3b* knockout mice also showed defects in blood differentiation, whereas zebrafish *sin3b* mutants showed locomotor defects (Cantor and David, 2017; Moravec et al., 2017). Although STRING analysis did not show any correlation with a specific phenotype (data available online), both g:Profiler analysis and the literature have reported that haploinsufficiency of *SIN3B* causes an ID and ASD phenotypes (Latypova et al., 2021).

There are several limitations to our study. First and foremost, limitations are the limited sample size and the lack of segregation and functional studies, especially for VUS variants and variants in poorly researched genes. Second, we focused only on non-synonymous coding variants, and by using a filtering process, we probably eliminated variants that were poorly covered or were present with low allelic frequency. For genes where protein function predictions and gene prioritization analysis results were not obtained, functional enrichment analysis with all ASD-associated genes should be performed in the future.

The abovementioned cases demonstrate that by performing WES analysis for patients with ASD without focusing on ASD-associated genes but including gene lists for genes that are functionally similar or linked to them, we yielded a higher diagnostic output, despite the limited clinical data. Reverse phenotyping by a clinician can then be performed to determine whether the variant in the analyzed gene is clinically relevant for the patient. In our study, focusing on the ultrarare variants or “single event” variants per gene regardless of their previously known molecular function was crucial for patients with complex phenotypes. This is illustrated by the high proportion of *de novo* variants identified as causative. Our study also demonstrates that most of the causative genes are involved in a syndromic form of ASD. In most patients, this is not the main feature, but rather part of a more complicated phenotype seen in these patients. Thus, classical genotype–phenotype analysis of WES data would be time-consuming, and perhaps in some cases, proper genotype–phenotype correlation would not be made due to several factors: a variable phenotype in specific disorders, overlapping clinical features, the revolving phenotype in small children, or reduced penetrance.

## Data Availability Statement

The datasets presented in this study can be found in online repositories. The names of the repositories and accession numbers can be found in the article. The datasets analyzed for this study can be found on the g:Profiler website (https://biit.cs.ut.ee/gplink/l/iGN_PmjmTv).

## Ethics Statement

The studies involving human participants were reviewed and approved by the Commission of the Republic of Slovenia for Medical Ethics. Written informed consent was obtained from the individual(s), and minor(s)’ legal guardian/next of kin, for the publication of any potentially identifiable images or data included in this article.

## Author Contributions

DK designed the study, analyzed the NGS data, performed the functional enrichment analysis and data interpretation, performed the validation by Sanger sequencing and the segregation analysis, and wrote the manuscript. MG and NR performed the NGS variant analysis workflow. MG participated in the chart and figure formation. IO and HG evaluated the patients. SS performed the validation by Sanger sequencing. MG, IO, SS, HG, PD, and NK revised the manuscript. All authors have read and approved the manuscript.

## Conflict of Interest

The authors declare that the research was conducted in the absence of any commercial or financial relationships that could be construed as a potential conflict of interest.

## Publisher’s Note

All claims expressed in this article are solely those of the authors and do not necessarily represent those of their affiliated organizations, or those of the publisher, the editors and the reviewers. Any product that may be evaluated in this article, or claim that may be made by its manufacturer, is not guaranteed or endorsed by the publisher.

## Footnotes

1
https://gene.sfari.org/tools/

2
https://franklin.genoox.com/clinical-db/home

3
https://string-db.org/

4
https://biit.cs.ut.ee/gprofiler/gost

5
spliceailookup.broadinstitute.org
